# Comparison of antibody-scTRAIL Fc fusion proteins with varying valency for EGFR and TRAIL receptors

**DOI:** 10.1038/s41598-025-00476-7

**Published:** 2025-05-06

**Authors:** Dennis Michler, Oliver Seifert, Klaus Pfizenmaier, Roland E. Kontermann

**Affiliations:** 1 Institute of Cell Biology and Immunology, University of Stuttgart, Allmandring 31, 70569 Stuttgart, Germany; 2 Stuttgart Research Center Systems Biology (SRCSB), University of Stuttgart, Nobelstraße 15, 70569 Stuttgart, Germany

**Keywords:** TNF-related apoptosis-inducing factor, TRAIL, ScTRAIL, Antibody fusion protein, Targeting, Cancer, Protein design, Recombinant protein therapy

## Abstract

Fusion proteins combining TNF-related apoptosis inducing ligand (TRAIL) and antibody building blocks have emerged as a strategy for the targeted treatment of cancer cells. Using a single-chain derivative of homotrimeric TRAIL (scTRAIL), several targeted and non-targeted scTRAIL fusion proteins of varying geometries and valencies for TRAIL receptors and target antigens, all comprising an Fc region, were generated. These fusion proteins comprised either 1 or 2 scTRAIL units, i.e. are tri- or hexavalent for TRAIL receptors and in the targeted versions, 1 or 2 binding sites for EGFR. These fusion proteins were analyzed for cell binding and cell death induction using the EGFR-expressing colorectal cancer cell lines Colo205 and HCT116. In line with previous findings, all fusion proteins that were hexavalent for TRAIL receptors exhibited a strongly increased cell killing activity compared to the trivalent ones. Interestingly, the fusion proteins comprising one scTRAIL unit, did not benefit from targeting to EGFR. In contrast, the hexavalent scTRAIL fusion proteins further benefited from EGFR targeting, resulting in an approximately 6- to 30-fold increase in cell killing. In summary, this study shed further light on the influence of geometry and valency of TRAIL fusion proteins and confirmed IgG-scTRAIL fusion proteins as highly potent cell death inducers.

**Supplementary Information:**

The online version contains supplementary material available at 10.1038/s41598-025-00476-7.

## Introduction

Antibody fusion proteins have emerged as a strategy for targeted delivery of therapeutic compounds^1^. In particular, antibody-cytokine fusion proteins have gained interest for cancer therapy, e.g. to deliver immunomodulatory activities^2–6^. The large family of cytokines also includes apoptosis-inducing proteins, such as TNF, FasL and TRAIL^7,8^. The preferential induction of apoptosis by TRAIL in tumor cells but not in normal cells has been identified as an advantage by providing a therapeutic window^9^. TRAIL can bind to two death receptors (TRAILR1 or DR4 and TRAILR2 or DR5) triggering apoptosis, but also to three decoy receptors (DcR1, DcR2, OPG) which can interfere with TRAIL-mediated apoptotic signals and contribute to TRAIL resistance of normal cells and cancer cells^10^. TRAIL is a homotrimeric protein and thus comprises three receptor binding sites, which are primarily expressed as membrane proteins, but can be cleaved from the membrane to form a soluble TRAIL^11^. Notably, it was found that, compared to membrane TRAIL, soluble TRAIL poorly triggers apoptosis through DR5, although both forms are capable of binding to DR4 and DR5, indicating that multiple interactions and higher order receptor clustering are required for activation^12,13^.

Various first- and second-generation TRAIL derivatives have been developed in the past years for cancer therapy. Dulanermin (AMG-951), a first-generation compound, is a soluble homotrimeric TRAIL variant which reached clinical phase 3 in combination with chemotherapy demonstrating synergistic activity, improved therapeutic activity and a favorable toxicity profile^14^. However, the development of dulanermin has not proceeded, one reason being a poor pharmacokinetic profile^15^. Nevertheless, a circularly permutated TRAIL derivative (CPT), aponermin, with a better stability and half-life has recently been approved in China for the treatment of multiple myeloma^16,17^. Eftozanermin alfa (ABBV-621) is a second-generation hexavalent TRAIL fusion protein utilizing fusion of a single-chain TRAIL derivative to a human Fc region to prolong half-life and to induce efficient receptor activation through multivalent TRAIL receptor binding^18^. Eftozanermin alfa has been tested in combination with venetoclax, a Bcl-2 inhibitor, in patients with AML and is currently in a phase 1 trial in combination with bortezomib and oral dexamethasone for the treatment of patients with multiple melanoma (NCT04570631)^19^.

TRAIL and TRAIL derivatives may benefit from further combination with a targeting moiety to allow a targeted delivery to tumor cells^20^. Development has especially advanced by converting the homotrimeric TRAIL into a single-chain derivative (scTRAIL)^21^. Using scTRAIL as a building block, various antibody-scTRAIL fusion proteins have been developed in the past years. These include scFv-scTRAIL derivatives that are trivalent for TRAIL receptors and various fusion proteins comprising a dimerization module and thus display two scTRAIL moieties and are therefore hexavalent for TRAIL receptors^21^. Hexavalent antibody-scTRAIL fusion proteins include Fc-less derivatives, such as Db-scTRAIL and EHD2-scTRAIL molecules, and Fc-comprising derivatives such as scFv-Fc-scTRAIL and IgG-scTRAIL fusion proteins^22–26^. These hexavalent antibody-scTRAIL fusion proteins are per se highly potent in activating TRAIL receptors, however, exhibit, dependent on the target specificity, compared to non-targeted versions increased cell death induction in vitro^22–24,26^. For example, an approximately 3- to 10-fold increased potency was found for EGFR-targeting scFv-Fc-scTRAIL fusion proteins^25^. To further investigate the influence of structure and composition of antibody-scTRAIL fusion proteins on cell killing activity, we generated various targeted and non-targeted scTRAIL fusion proteins with different geometries and valencies for TRAIL receptors and the target antigen EGFR, all comprising an Fc region. EGFR was chosen as target antigen since EGFR is a validated target for tumor therapy and extensive data on EGFR-targeting scTRAIL fusion proteins have been generated in the past^22–26^. These molecules were analyzed for target cell binding and cell death induction using two different CRC cancer cell lines sensitive to TRAIL-mediated apoptosis induction. Our study highlights the importance of valency and showed that the position of the antibody and scTRAIL units had only minor effects on cell death induction, providing a rational for the generation of antibody-scTRAIL fusion proteins.

## Results

### Generation of antibody-scTRAIL fusion proteins

**Fig. 1:**
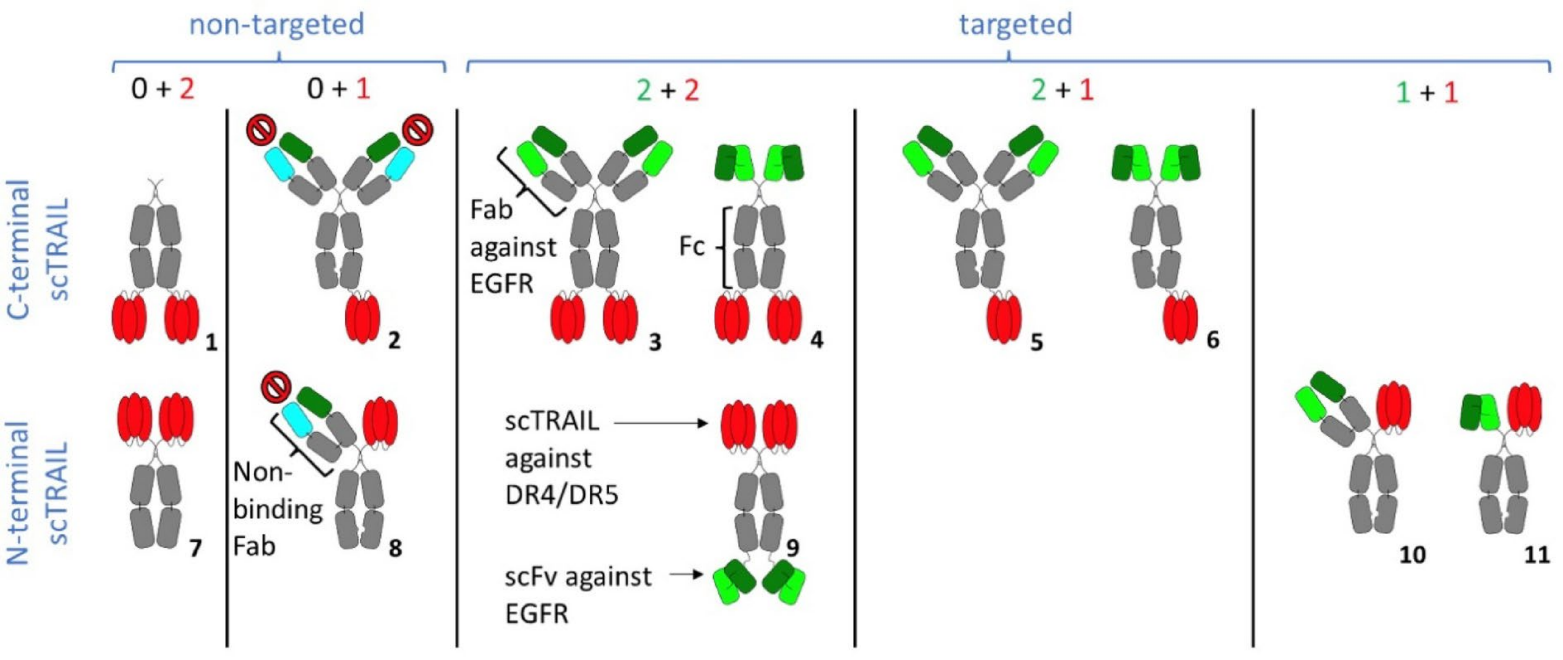
Overview of scTRAIL fusion proteins with different numbers of scTRAIL units (red) and antigen-binding site for EGFR (light and dark green). Constant antibody regions are shown in grey, the VL of a dummy antibody is shown in cyan. The first number refers to the number of antigen binding sites, the second number to the number scTRAIL units. The following molecules have been generated: (1) Fc-scTRAIL (0 + 2); (2) DIgG-Fc-scTRAIL (0 + 1); (3) IgG-scTRAIL (2 + 2); (4) scFv-Fc-scTRAIL (2 + 2); (5) IgG-scTRAIL (2 + 1); (6) scFv-Fc-scTRAIL (2 + 1); (7) scTRAIL-Fc (0 + 2); (8) DFab-scTRAIL-Fc (0 + 1) 9); scTRAIL-Fc-scFv (2 + 2); 10) Fab-scTRAIL-Fc (1 + 1); 11) scFv-scTRAIL-Fc (1 + 1).

Various targeted and non-targeted scTRAIL fusion proteins with different geometries and varying valencies for TRAIL receptors and the target antigen EGFR were generated (Fig. 1). Non-targeted scTRAIL fusion proteins (ntTFPs) consist of two scTRAIL moieties fused to either the N- or C-terminus of a human Fc region (0 + 2; molecules #1 and #7), thus are hexavalent for TRAIL receptors. Targeted scTRAIL fusion proteins (tTFPs) contain one or two antigen-binding sites directed against EGFR. Fusion of scTRAIL to the C-terminus of an IgG or a scFv-Fc, or to the N-terminus of a Fc-scFv resulted in molecules that were bivalent for EGFR and hexavalent for TRAIL receptors (2 + 2; molecules #3, #4, #9). Furthermore, by using a heterodimerizing knobs-into-holes Fc region, tTFPs with only one scTRAIL moiety were generated with either two binding sites for EGFR (2 + 1; molecules #5, #6) or one binding site for EGFR (1 + 1; molecules #10 and #11). In addition, we generated two control ntTFPs with one or two dummy binding sites (0 + 1; molecules #2 and #8) by replacing the EGFR-specific light chain with an irrelevant one. All fusion proteins were produced in HEK293-6E and purified by protein A chromatography with yields between 1 and 14 mg protein per liter of supernatant (Suppl. Table 1). SDS-PAGE confirmed purity, dimer formation under non-reducing conditions and the presence of all expected chains under reducing conditions (Suppl. Figure 1). Purity and integrity were further confirmed by size-exclusion chromatography (Suppl. Figure 2, Suppl. Table 1).

### Binding to EGFR and TRAIL receptor 2 (DR5)

**Fig. 2:**
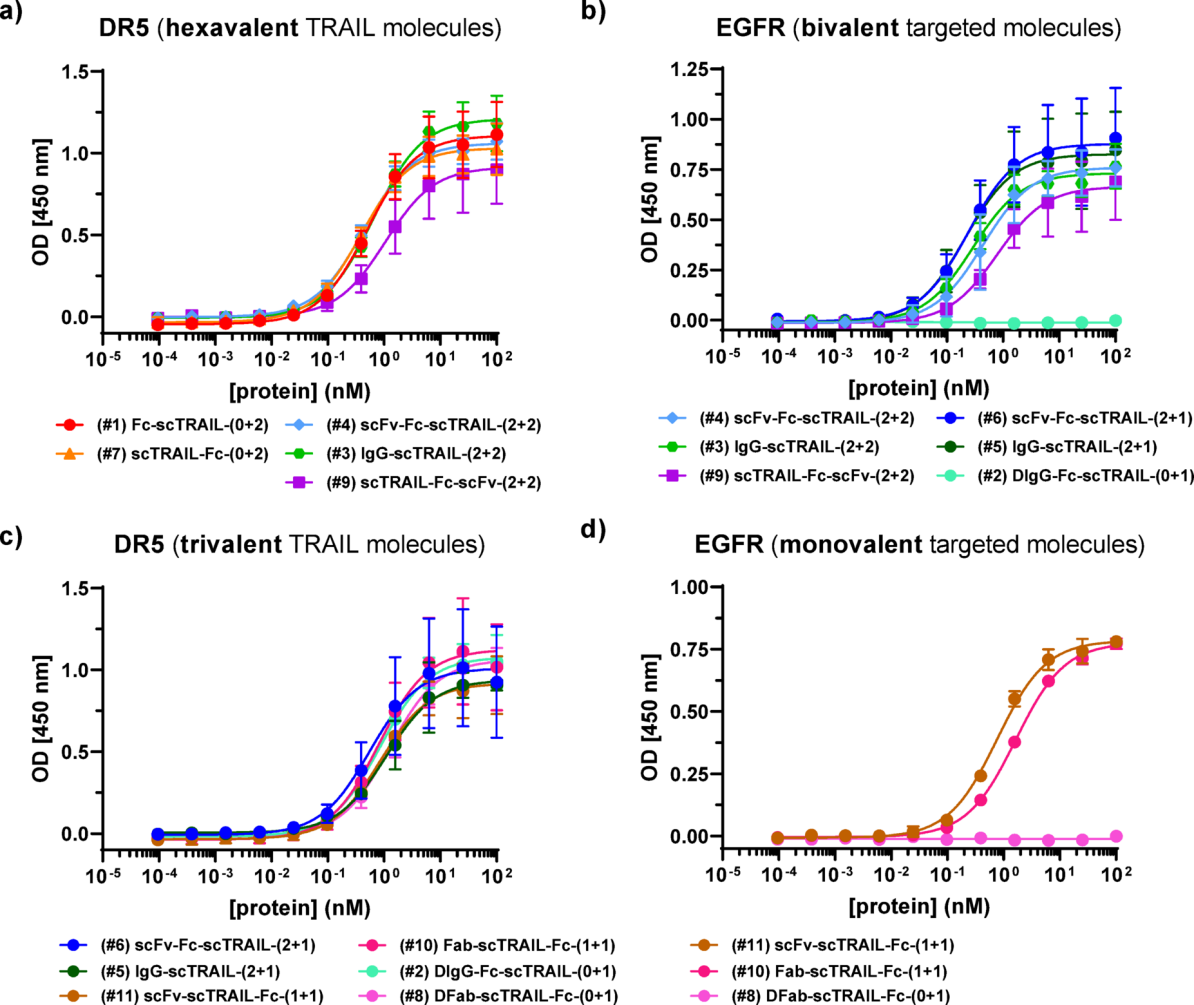
Binding of antibody-scTRAIL fusion proteins to immobilized DR5 and EGFR analyzed by ELISA. **(A)** Hexavalent TRAIL molecules on DR5. **(B)** Trivalent TRAIL molecules on DR5. **(C)** Bivalent targeted molecules to EGFR. **(D)** Monovalent targeted molecules to EGFR. scTRAIL-antibody fusion proteins were titrated at a 1:4 dilution between concentrations starting at 100 nM, coated with DR5- or EGFR-moFc (0.3 µg/well), detected with HRP-conjugated anti-human Fc antibody. *n* = 3, mean ± SD.

The binding of the antibody-scTRAIL fusion proteins was analyzed by ELISA (Fig. 2). All antibody-scTRAIL fusion proteins showed a concentration-dependent binding to DR5 and EGFR. All fusion proteins with two scTRAIL moieties, except scTRAIL-Fc-scFv (2 + 2, #9), exhibited EC_50_ values in the sub-nanomolar range (between 383 and 655 pM) for DR5 binding, with the lowest EC_50_ values observed for scTRAIL-Fc (0 + 2; #7) (Fig. 2a, c; Table 1). ScTRAIL-Fc-scFv (#9) exhibited an EC_50_ value of 1.1 nM, which was in the range of trivalent TRAIL molecules with EC_50_ values of around 1 nM (753 to 1283 pM) (Table 1). All antibody fusion proteins bound to EGFR with sub-nanomolar or single-digit nanomolar EC_50_ values in ELISA. The strongest EGFR binding in ELISA was observed for IgG fusion-proteins (#3, #5) with EC_50_ values between 210 and 290 pM.

**Table 1:** Binding EC_50_ values (ELISA and flow cytometry) and cytotoxic effects of the scTRAIL-antibody fusion proteins to their target antigens DR5 and EGFR and EGFR-positive cell lines Colo205 and HCT116. *n* = 3; mean ± sd; -: not performed.

Moieties	Mole- cule	Format	ELISA		Cell binding		Cytotoxicity			
			DR5	EGFR	Colo205	HCT116	Colo205		HCT116	
Fab/scFv + scTRAIL			EC_50_ [pM]	EC_50_ [pM]	EC_50_ [pM]	EC_50_ [pM]	EC_50_ [pM]	Maximal killing [%]	EC_50_ [pM]	Maximal killing [%]
**0 + 2**	#1	Fc-scTRAIL	493 ± 53	-	5,173 ± 3,085	3,850 ± 990	36 ± 4	100	71 ± 30	75
**0 + 2**	#7	scTRAIL-Fc	383 ± 64	-	4,411 ± 1,725	6,177 ± 1,027	52 ± 4	100	177 ± 24	75
**2 + 2**	#3	IgG-scTRAIL	655 ± 145	291 ± 58	86 ± 20	129 ± 25.8	6 ± 1	100	6 ± 2	75
**2 + 2**	#4	scFv-Fc-scTRAIL	434 ± 22	496 ± 232	244 ± 141	351 ± 46	6 ± 1	100	8 ± 2	75
**2 + 2**	#9	scTRAIL-Fc-scFv	1,069 ± 124	768 ± 142	997 ± 349	633 ± 134	9 ± 2	100	12 ± 6	75
**2 + 1**	#5	IgG-scTRAIL	1,268 ± 456	211 ± 34	51 ± 20	352 ± 274	1,756 ± 1076	25	256 ± 142	50
**2 + 1**	#6	scFv-Fc-scTRAIL	753 ± 71	2,344 ± 16	73 ± 22	188 ± 147	1,536 ± 1155	38	499 ± 276	55
**1 + 1**	#11	scFv-scTRAIL-Fc	883 ± 381	767 ± 28	258 ± 123	1,492 ± 999	418 ± 211	50	472 ± 60	60
**1 + 1**	#10	Fab-scTRAIL-Fc	929 ± 185	1,641 ± 144	255 ± 23	746 ± 65	1,385 ± 449	50	727 ± 43	60
**0 + 1**	#2	DIgG-Fc-scTRAIL	1,283 ± 295	-	3,153	72,000	601 ± 75	100	844 ± 445	70
**0 + 1**	#8	DFab-scTRAIL-Fc	853 ± 208	-	5,680	18,000	139 ± 42	100	264 ± 141	70

### Binding to EGFR-expressing cell lines

**Fig. 3:**
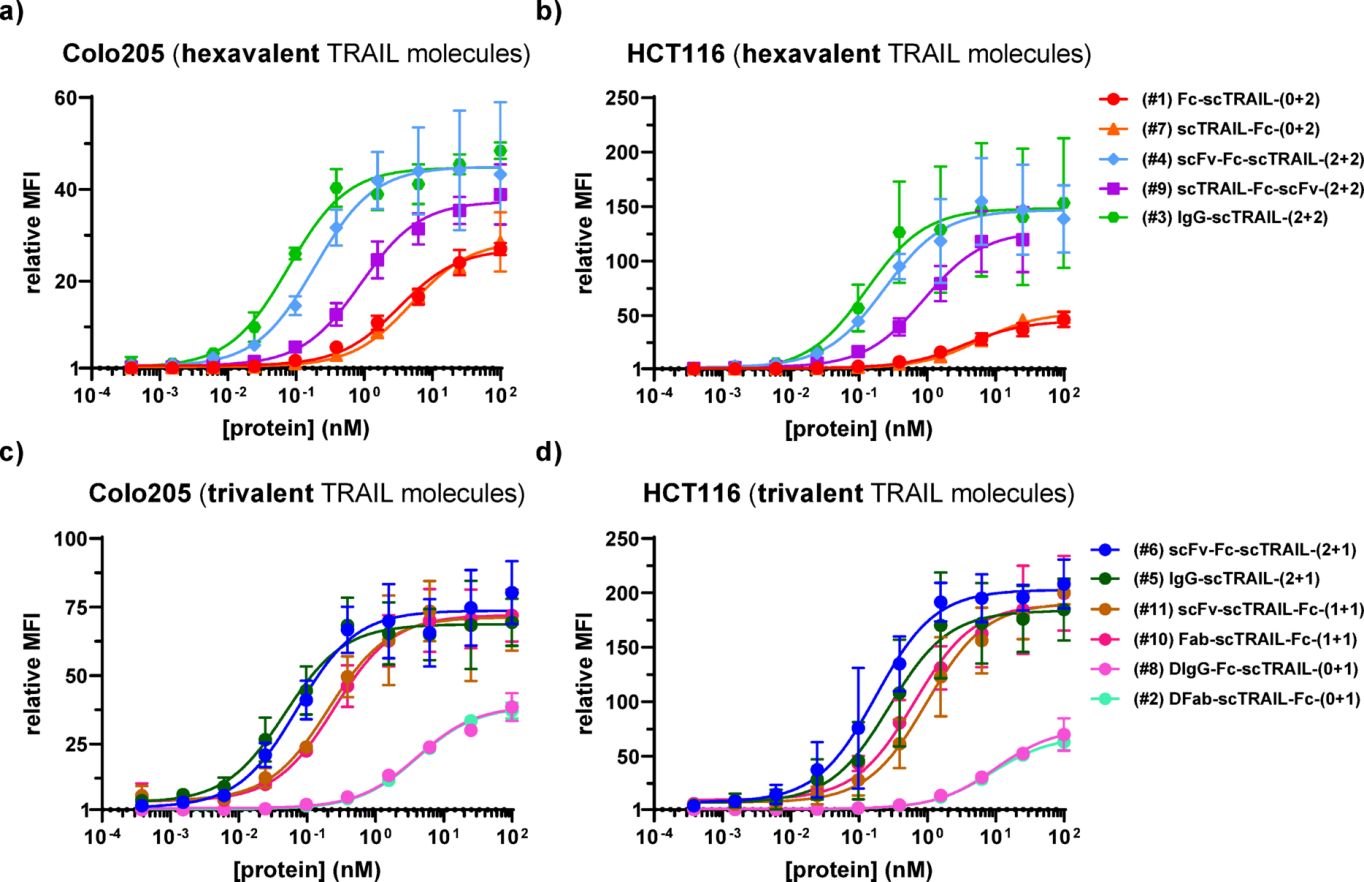
Binding of antibody-scTRAIL fusion proteins to Colo205 and HCT116 cells analyzed by flow cytometry. **(A)** Hexavalent TRAIL molecules on Colo205. **(B)** Trivalent TRAIL molecules on Colo205. **(C)** Hexavalent TRAIL molecules on HCT116. **(D)** Trivalent TRAIL molecules on HCT116. scTRAIL-antibody fusion proteins were titrated at a 1:4 dilution between concentrations starting at 100 nM, 100,000 cells/well, detection with PE-conjugated anti-human Fc antibody. *n* = 3, mean ± SD.

The binding of the different fusion proteins to the EGFR-expressing human colorectal cancer cell lines Colo205 and HCT116 were investigated by flow cytometry (Fig. 3). Both cell lines express EGFR (~ 17,000 receptors/cell for Colo205, ~ 23,000 receptors/cell for HCT116) and express low levels of TRAILR1 (DR4) (Colo205 ~ 500 receptors/cell, HTC116 ~ 7,500 receptors/cell) and between 1,000 and 2,000 receptors/cell for TRAILR2 (DR5), TRAILR3 (DcR1) and TRAILR4 (DcR2)^25^. All molecules showed binding to both cell lines in a concentration-dependent manner. In general, tTPF molecules containing 1 or 2 EGFR binding sites bound to both cell lines with lower EC_50_ values than the ntTFPs (Suppl. Table 3a, b). Furthermore, the ntTFPs reached lower maximal MFIs compared to the tTFPs (Fig. 3). The best binding with the lowest EC_50_ values was observed for the IgG-scTRAIL fusion proteins (2 + 2, #3 and 2 + 1, #5) with EC_50_ values of 86 and 51 pM on Colo205 cells and 129 and 352 pM on HCT116 (Fig. 3; Table 1).

### Cytotoxicity against EGFR-expressing cell lines

**Fig. 4:**
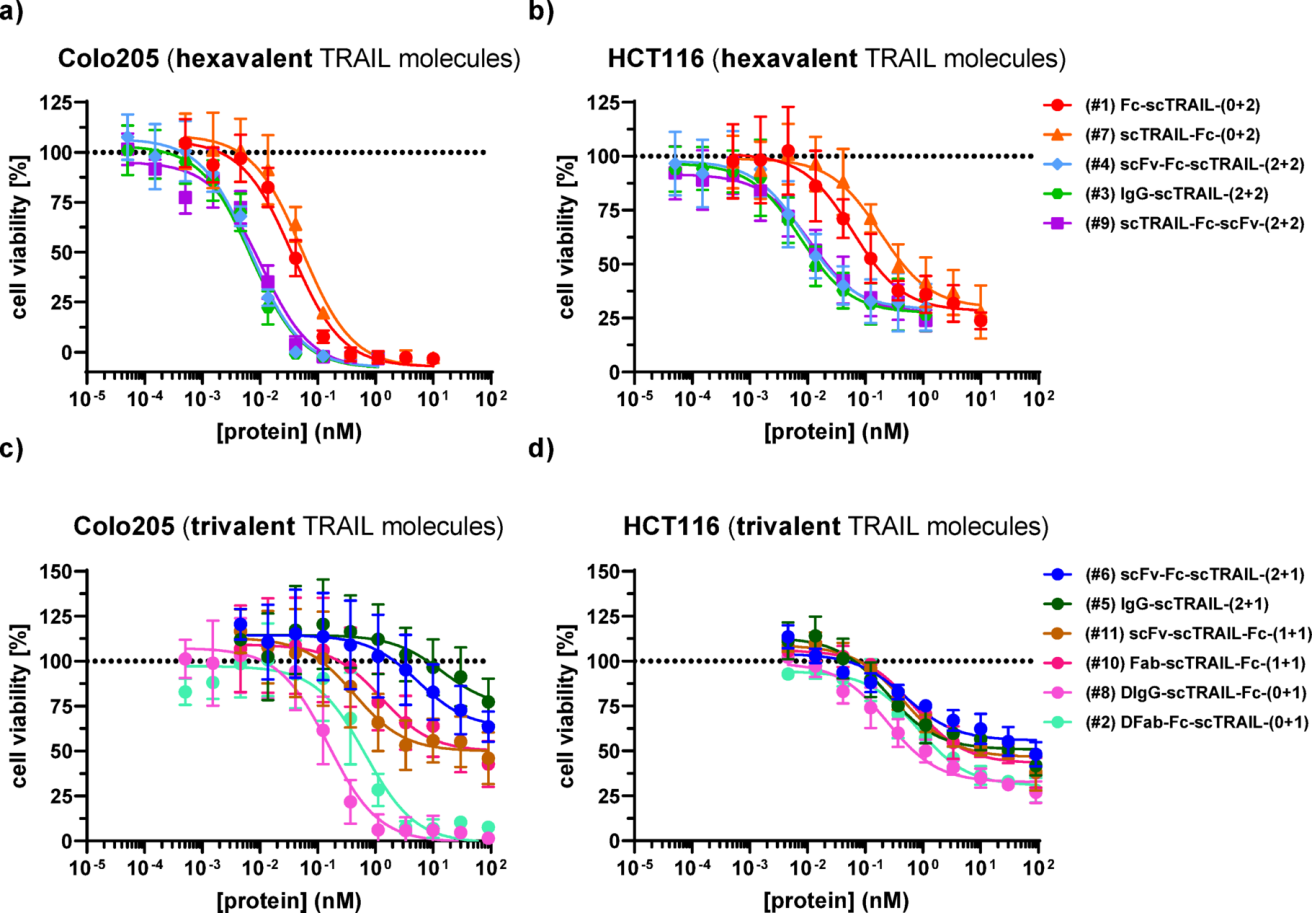
Cell death induction assay of hexavalent and trivalent TRAIL molecules on Colo205 and HCT116 cells. **(A)** Hexavalent TRAIL molecules on Colo205. **(B)** Trivalent TRAIL molecules on Colo205. **(C)** Hexavalent TRAIL molecules on HCT116. **(D)** Trivalent TRAIL molecules on HCT116. scTRAIL-antibody fusion proteins were titrated at a 1:3 dilution between concentrations starting at 1 nM (2 + 2 formats) or 10 nM (0 + 2 formats), 50,000 cells/well, detection by crystal violet staining. *n* = 3, mean ± SD.

To determine the cell killing activity, Colo205 and HCT116 were incubated with the scTRAIL fusion proteins for 18 h. Killing of both cell lines was induced by all antibody-scTRAIL fusion proteins in a concentration-dependent manner, but with varying potency and efficacy. Generally, fusion proteins with 2 scTRAIL moieties (#1, #7, #3, #4, #9) achieved a maximum killing of 100% for Colo205 and 75% for HCT116 at the highest concentrations and showed lower EC_50_ values than molecules with only one scTRAIL moiety (#5, #6, #10, #11) maximum killing of 25 to 50% for Colo205, 50 to 60% for HCT116 (Table 1; Fig. 4, Suppl. Table 3 g, h). Very strong killing with EC_50_ values in the range of 6 to 12 pM was observed for all 2 + 2 tTFPs (#3, #4, #9), followed by the 0 + 2 ntTFPs (#1, #7) with EC_50_ values in the range of 36–177 pM. All targeted 2 + 1 and 1 + 1 fusion proteins with 1 scTRAIL moiety (#5, #6, #10, #11) displayed reduced killing activity with EC_50_ values between 418 and 1,756 pM (Suppl. Table 3 g, h). Unexpectedly, the non-targeted (dummy) scTRAIL molecules (#2, #8) comprising a single trimeric scTRAIL moiety showed a similar or even better killing activity as compared to the targeted 2 + 1 and 1 + 1 tTFPs (#5, #6, #10, #11). A comparison of the dummy 0 + 1 Fab-scTRAIL fusion protein (#8) with the corresponding EGFR-targeting 1 + 1 Fab-scTRAIL (#10) incubated with or without the EGFR antibody cetuximab showed that blocking the binding site on EGFR improved maximal killing from 75 to 100%, although not fully reaching the activity of the dummy 0 + 1 Fab-scTRAIL (#8) (Suppl. Figure 3).

### Membrane expression of EGFR and death receptors

Using HCT116 cells we analyzed the membrane expression of EGFR and the death receptors by immunofluorescence staining of fixed cells. EGFR could be localized throughout the membrane although at different intensities (Suppl. Figure 4). In contrast, DR4 and DR5, both detected with the same secondary antibody, were mainly found in fewer and larger clusters, most of them in proximity to EGFR.

### Caspase-3/7 activity

**Fig. 5:**
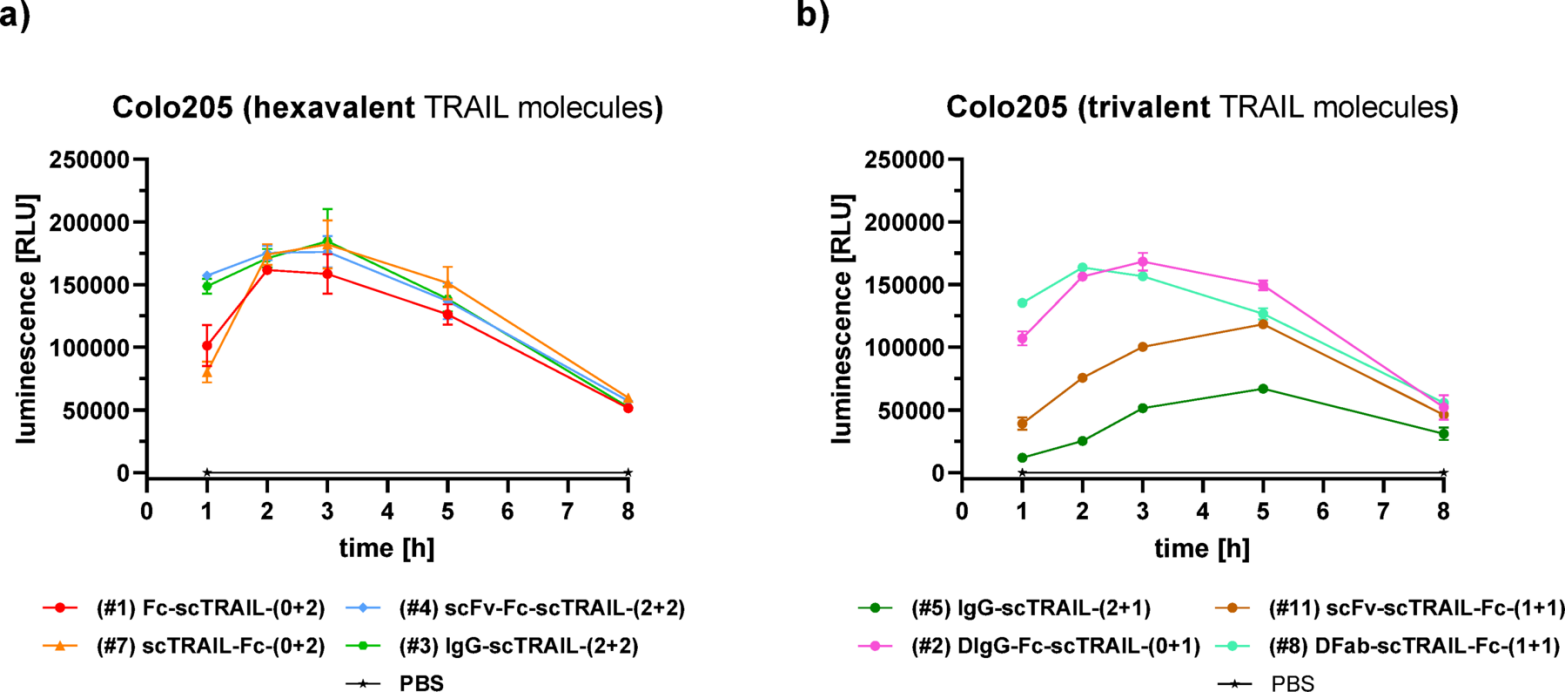
Caspase-3/7-assay of hexavalent and trivalent TRAIL molecules on Colo205. **(A)** Hexavalent TRAIL molecules on Colo205. **(B)** Trivalent TRAIL molecules on Colo205. scTRAIL-antibody fusion proteins were added at 1 nM final concentration to 15,000 cells/well, luminescence signal is proportional to the amount of caspase activity. *n* = 1, mean of duplicates.

The kinetics of apoptosis induction was analyzed by measuring the caspase-3/7 activity using Colo205 cells incubated with selected scTRAIL fusion proteins for up to 8 h (Fig. 5). Most of the molecules reached a maximal caspase activity after 2–3 h, with the exception of the 2 + 1 and 1 + 1 tTFPs (#5, #6, #10, #11), which reached a maximum at 5 h and also had lower maximal RLUs. Of note, the dummy 0 + 1 molecules (#2, #8) exhibited a similar kinetic and reached similar maximal RLUs as the 0 + 2 and 2 + 2 molecules (#1, #7, #3, #4, #9).

### Comparison of cell binding and killing activity

**Fig. 6:**
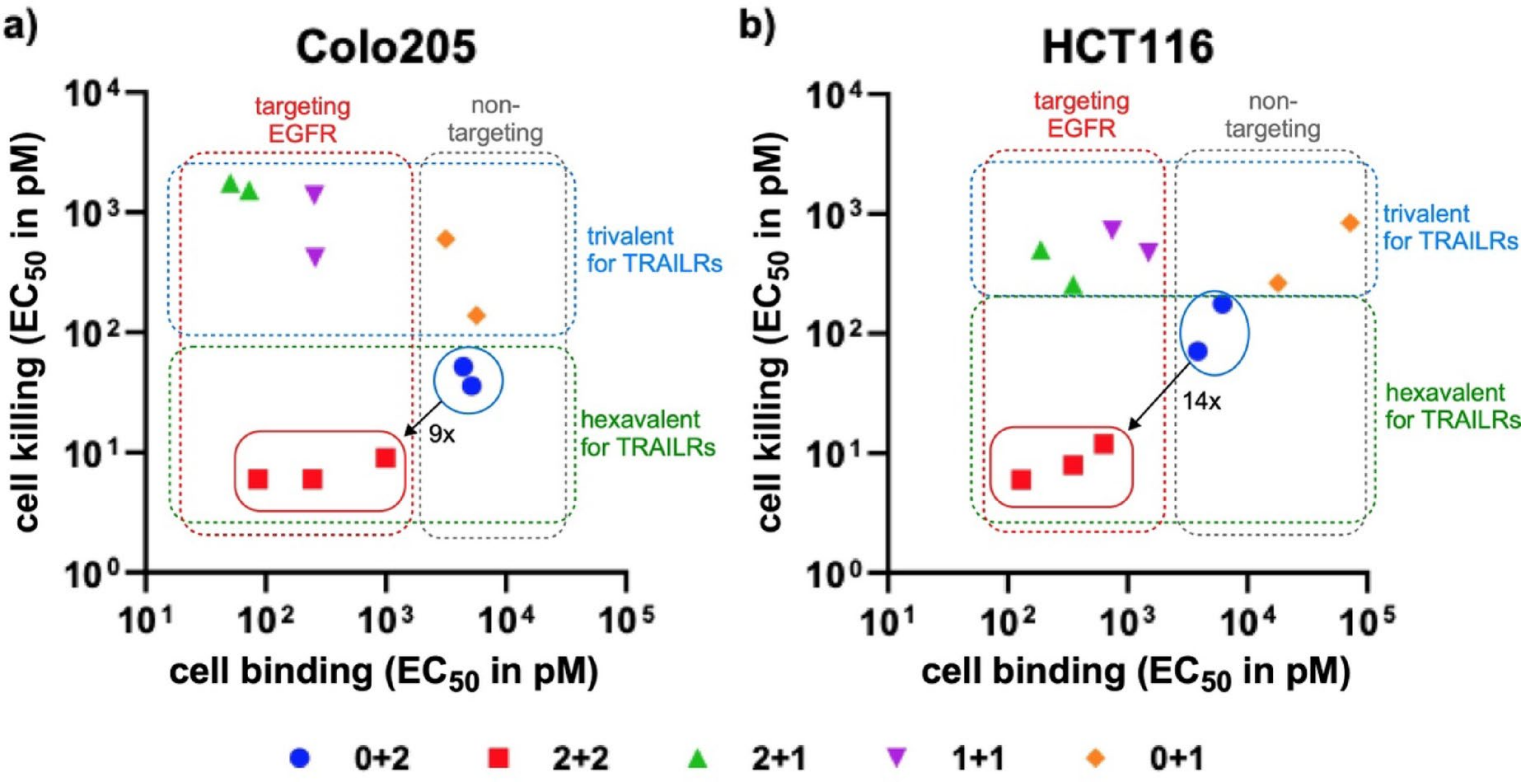
Correlation between cell binding and killing. Cell binding and cell killing activity of the different scTRAIL fusion proteins are plotted, demonstrating the beneficial effects of targeting hexavalent scTRAIL fusion proteins on cell killing activity.

A comparison of cell binding and cell killing activity of the different tTFPs and ntTFPs showed that all trivalent scTRAIL fusion proteins exhibited a cell killing with EC_50_ values above those of the hexavalent fusion proteins, irrespective of whether they were targeted or non-targeted, i.e. irrespective of cell binding. Notably, a beneficial effect of increased cell binding due to EGFR targeting was observed for the hexavalent fusion proteins (#3, #4, #9). Here, on average, cell killing increased approximately 9-fold for Colo205 and 14-fold for HCT116 (Fig. 6).

## Discussion

TRAIL molecules are capable of binding to both death receptors (DR4, DR5), irrespective whether expressed as membrane-bound or soluble ligand. However, activation of DR5 requires receptor clustering by membrane-bound TRAIL for potent activation, whereas DR4 is activated by both membrane-bound and soluble TRAIL^12,13^. Conceptually, it has been established that membrane-bound TRAIL can be mimicked by hexavalent TRAIL fusion proteins or by mediating membrane presentation of trivalent TRAIL by binding a surface antigen, e.g. using an antigen-binding site of an antibody^20,27^.

In the present study, we focused on Fc-fusion proteins that use a silent Fc to exclude Fc-mediated binding while maintaining FcRn-mediated recycling and, thus a prolonged serum half-life^24,28^. Using different geometries of these Fc-fusion proteins we confirmed the potent death induction by hexavalent scTRAIL fusion proteins (#1, #7, #3, #4, #9) compared to trivalent scTRAIL molecules (#5, #6, #10, #11). Thus, non-targeted Fc-scTRAIL and scTRAIL-Fc, with two scTRAIL units fused to either the C- or N-terminus of a human γ1 Fc region (#1, #7) induced cell death of Colo205 and HCT116 cells, expressing both death receptors, induced already very potent cell killing. Comparing the two non-targeted 0 + 2 Fc fusion proteins (Fc-scTRAIL #1, scTRAIL-Fc #7), we found a slightly better activity (1.4 to 2.5-fold) for the Fc-scTRAIL molecule (#1), i.e. with the scTRAIL at the C-terminus of the Fc-region. This was also seen for the 2 + 2 targeted versions, which also contain scFv units for EGFR targeting (4, #9), with a slightly better activity for scFv-Fc-scTRAIL (#4). This might be due to differences in the flexibility and distance of the scTRAIL units. Nevertheless, these differences are minor and non-targeted scTRAIL-Fc fusion proteins, with the scTRAIL moiety fused to the N-terminus of the Fc-region (eftozanermin alfa, ABBV-621) has already enter clinical studies. Eftozanermin alfa has been reported to kill 36% of 126 cancer cell lines tested in vitro with subnanomolar EC_50_values, similar to our scFv-Fc fusion protein^18^. In a clinical phase 1 study eftozanermin alfa was used as a combination therapy with the BCL-2 selective inhibitor venetoclax to activate the intrinsic and extrinsic apoptotic pathways and to improve clinical efficacy and was proven to be well tolerated^19^.

In vitro, the efficacy of non-targeted hexavalent scTRAIL fusion proteins (1, #7) was further increased by 6- to 12-fold on Colo205 and 9- to 30-fold on HCT116 by adding an EGFR-targeting moiety (#3, #4, #9). Interestingly, the fusion proteins comprising one scTRAIL unit did not benefit from targeting to EGFR, regardless of whether one or two EGFR-targeting moieties were present. Even more, two different dummy 0 + 1 fusion proteins (#2, #8) were found to be more potent than the corresponding 2 + 1 (#5, #6) and 1 + 1 fusion proteins (#10, #11), indicating that binding to a tumor-associated antigen can also interfere with cytotoxic activity, pointing to a detargeting effect. This might be due to the fact that EGFR is expressed throughout the plasma membrane while DR4 and DR5 are mainly located in clusters. Binding of fusion proteins comprising one scTRAIL unit to EGFR located farther away from death receptors might interfere with death receptor binding and thereby reduce cytotoxic activity. In constrast, molecules with two scTRAIL units and thus being hexavalent for TRAIL receptors might benefit from an increased avidity and thus preferentially bind to TRAIL receptors, which might be further enhanced by binding to nearly EGFR. This finding is in contrast to that described for an anti-CD19 IgG-scTRAIL heterodimeric fusion protein containing one scTRAIL moiety, for which potent killing of CD19-positive tumor cells was observed in vitro and in vivo, whereas a control fusion protein targeting HER2 was inefficient^29^. This might be due to the preferential activation of DR5 in a target-dependent manner and thus depend on the target cell.

Differences on cell killing dependent on the target have previously been described for scFv-Fc-scTRAIL fusion proteins targeting EGFR, HER2, HER3, and EpCAM^25^. Here, a targeting effect was found for fusion proteins targeting EGFR, HER3 and EpCAM, but not for HER2, using the same cell lines as in the present study. Furthermore, a comparison of two HER3-targeting antibodies binding to different domains of HER3 revealed a stronger killing activity for a fusion protein targeting domains 3 and 4 (antibody 3–43) than an antibody targeting domain 1 (derived from MM-121). In addition, other effects such as trafficking and processing of the fusion proteins and its targets through target-mediated internalization might affect the cytotoxic activity. It is well-known that anti-EGFR antibodies such as cetuximab are rapidly internalized upon target binding which leads to EGFR and antibody degradation^30^. In this context, the affinity and avidity (valency) for EGFR might further affect internalization and thus TRAIL receptor activation. Thus, as shown for HER2 antibodies, a reduced antibody internalization and catabolism was demonstrated for lower affinity antibodies and for monovalent antibody derivatives exhibiting a reduced binding kinetic compared to a bivalent antibody^31,32^. Similarly, DR4 and DR5 are internalized upon ligand binding^33^. Of note, ligand-activated death receptors can signal through membrane-associated and intracellular complexes^34^. Thus, the beneficial effects of targeting tri- and hexavalent scTRAIL fusion proteins might therefore not only depend on the target and the targeting antibody, but also on the cellular context and cellular processes.

In summary, format and geometry (arrangement of binding sites) seem to have little effect on target cell killing activity. The greatest effect on target cell killing is obtained using targeted fusion proteins with hexavalent TRAIL receptor binding. This is in line with previous findings for EGFR-targeting diabody-scTRAIL fusion proteins (Db-scTRAIL) and dimeric EHD2-scTRAIL fusion proteins, all exhibiting very strong tumor cell killing activity with subnanomolar EC_50_values, although different pharmacokinetic properties^22–24^. Studies using different antibodies against EGFR and other tumor targets, to generate targeted hexavalent scTRAIL fusion proteins, will shed further light on the relevance of targeting on TRAIL’s antitumoral activity.

## Materials and methods

### Cell lines and materials

HEK293-6E Colo205 and HCT116 cell lines were purchased from ATCC. HEK293 were cultured in RPMI 1640 medium (Thermo Fisher) supplemented with 5% fetal bovine serum (FBS, Sigma Aldrich). Colo205 and HCT116 were cultured in RPMI 1640 medium supplemented with 10% FBS. For the production of recombinant proteins, HEK293-6E cells were cultivated in FreeStyle™ F17 expression medium (Thermo Fisher) supplemented with GlutaMAX-I™ (Thermo Fisher, 4 mM) and Kolliphor^®^ P 188 (Sigma-Aldrich, 0.1%). All cell lines were incubated in an orbital shaker at 37 °C, 5% CO_2_. Polyethyleneimine (PEI) was purchased from Sigma Aldrich and tryptone N1 was purchased from Organotechnie.

### Production and purification of Recombinant proteins

All antibody-scTRAIL fusion proteins were cloned into the pSecTagAL1 expression vector. A humanized version (hu225) of cetuximab was used as the EGFR targeting moiety^35^. The single-chain TRAIL moiety (118-1), which contains a 1 amino acid linker between the TRAIL units starting at residue 118, was used^24^. For transient transfection, HEK293-6E cells were incubated at 37 °C, 5% CO_2_ and 170 rpm until a cell density of approximately 10^6^ cells/mL was reached. PEI and the plasmid DNA for the desired antibody chain(s) were then mixed in a 2:1 ratio. This mixture was pre-incubated with F17 + + medium and added to the cells after 15 min. After 24 h incubation time the F17 + + medium was supplemented with 20% TN1 and 100 nM ZnCl_2_ (2.5% and 0.05% of the culture medium volume). After 96 more hours, the recombinant proteins were harvested by centrifugation and purified by protein A chromatography. Antibody-scTRAIL fusion proteins were eluted with 3.5 M MgCl_2_ and dialyzed against PBS at 4 °C overnight. SDS-PAGE was performed to verify the presence and purity of antibody chains produced (3 µg for non-reducing/6 µg for reducing conditions). Proteins were separated in 12% polyacrylamide gels and stained with Coomassie Brilliant Blue G-250. For SEC analysis samples were injected onto a Yarra™ 3 μm SEC-2000 column using 0.1 M Na_2_HPO_4_/NaH_2_PO_4_, 0.1 M Na_2_SO_4_, pH 6.7 as mobile phase at a flow rate of 0.5 ml/min. Aliquots were stored at – 80 °C.

### ELISA

ELISA high-binding plates were coated with TRAIL-R2-moFc or EGFR-moFc (3 µg/ml) overnight at 4 °C. Residual binding sites were blocked with 2% MPBS (150 µl/well, 2 h, RT). Antibody-scTRAIL fusion proteins were diluted in MPBS and titrated 1:4 in duplicates starting at 100 nM (100 µl/well, 1 h, RT). Bound proteins were detected with anti-huFc-HRP antibody (A0170, Sigma Aldrich, 100 µl/well, diluted 1:5,000 in MPBS, 1 h, RT) using 3,3′,5,5′-tetramethylbenzidine (TMB) as substrate (0.1 mg/ml TMB, 100 mM sodium acetate buffer, pH 6.0, 0.006% H_2_O_2_). After stopping the reaction with 50 µl of 1 M H_2_SO_4_, the absorbance was measured at 450 nm. Between each step and before detection, the plates were washed twice with PBS-T (PBS + 0.005% Tween-20) and once with PBS.

### Flow cytometry

10^5^ Colo205 or HCT116 cells/well were seeded into a 96-well U-bottom plate in RPMI + 10% FBS. Antibody-scTRAIL fusion proteins were diluted in PBA and titrated 1:4 in duplicates starting at 100 nM (100 µl/well, 1 h, 4 °C). For detection the anti-huFc-PE antibody was used (12–4998-82, Thermo Fisher, 100 µL/well, diluted 1:500 in PBA, 1 h, 4 °C). Finally, 100 µl of PBA was added to the cells and the fluorescence signal was measured using the MACSQuant VYB flow cytometer. Between each step, the cells were washed three times by alternating resuspension with 150 µl PBA and centrifugation (1,500 rpm, 4 °C, 3 min).

### Cell death assays

Colo205 (5 × 10^4^ cells/well) or HCT116 cells (1.5 × 10^4^ cells/well) were seeded in F-bottom 96-well plates and incubated in medium (100 µl/well, RPMI + 10% FBS + P/S) at 37 °C, 5% CO_2_ for 24 h. Next, a dilution series (1:3 in RPMI + 10% FBS + P/S) of the antibody-scTRAIL fusion proteins was transferred to the cells in duplicates (100 µl/well) and incubated at 37 °C, 5% CO_2_ for 18 h. As a control, cells were treated with 100 µl of medium only (RPMI + 10% FBS + P/S) and a death control with 15 µl Triton X-100, both in duplicates. Viable cells were detected by crystal violet staining.

### Immunofluorescence staining

Glass coverslips were coated with collagen-R in PBS overnight. After two washes with 1 ml PBS, 10^5^ cells were seeded and incubated at 37 °C, 5% CO_2_ for 24 h. After incubation, the cells were washed once with 1 ml of pre-warmed PBS (+ CaCl₂ and MgCl₂) and fixed with 1 ml of 4% paraformaldehyde (PFA) solution for 15 min. Afterwards the cells were washed three times with 1 ml PBS and a blocking step was performed using 1 ml PBS containing 5% goat serum for 1.5 h. The primary antibody was diluted in blocking solution and incubated for 1.5 h (anti-TRAIL-R1 (D9S1R, CST): 1:800, anti-TRAIL-R2 (D4E9, CST): 1:100, anti-EGFR-FITC (sc-120, Santa Cruz): 1:50). After primary antibody incubation, samples were washed three times with 1 ml PBS. Secondary antibody (anti-rabbit Alexa Fluor 647 (A-21245, Thermo Fisher) was diluted 1:500 and incubated in 500 ml blocking solution for 1 h. DAPI (1:5,000) was added in the last 15 min. Next, cells were washed three times with 1 ml PBS. Finally, cells were mounted onto glass slides with ProLong Gold and allowed to cure overnight. Imaging was performed using a Zeiss LSM 980 with Airyscan 2 equipped with a 63x/1.4 oil DIC. Images were acquired using ZEN software.

### Caspase − 3/7 activity assays

Colo205 cells (15,000 cells/well) were seeded in 100 µl of medium (RPMI + 10% FBS, P/S) in a F-bottom 96-well plate and incubated at 37 °C, 5% CO_2_ for 24 h. Next day, the medium was removed by aspiration and replaced with 60 µl fresh medium. The antibody of interest was then transferred to the cells in duplicate (20 µl, 1 nM for hexavalent and 90 nM for trivalent TRAIL molecules) and incubated at 37 °C, 5% CO_2_ for 1, 2, 3, 5, 8, and 20 h. For negative control, only PBS was added to the cells. After each incubation period, the corresponding plate was removed from the incubator and 20 µl of Caspase-Glo-3/7-reagent was added. After 30 min the luminescence signal was detected, which correlates with the caspase − 3/7 activity.

### Statistics

All data are presented as mean ± SD of at least three independent experiments. The measured value of an experiment is always the mean value of a duplicate. Only caspase − 3/7 activity assays and the blocking experiment with cetuximab were performed only once. Pairwise and multiple comparisons were performed using unpaired *t* test (two-tailed) and one-way ANOVA, respectively, followed by Tukey *post hoc* test, respectively (GraphPad Prism software). *P* < 0.05 was considered statistically significant.

## Electronic supplementary material

Below is the link to the electronic supplementary material.

Supplementary Material 1

## Data Availability

All the data generated and/or analyzed during this study are included in this research article and its supplementary information files.

## Abbreviations

DR: Death receptor
EGFR: Epidermal growth factor receptor
EpCAM: Epithelial cell adhesion molecule
FasL: Fas ligand
Fc: Fragment crystallizable
HER2/3: Human epidermal growth factor receptor 2/3
HMW: High molecular weight
MPBS: Milk powder phosphate buffered saline
ntTFPs: Non-targeted scTRAIL fusion proteins
PBA: PBS (phosphate buffered saline) + BSA (bovine serum albumin) + azide
RLU: Relative light units
scFv: Single-chain Fragment variable
scTRAIL: Single-chain tumor necrosis factor-related apoptosis-inducing ligand
SEC: Size exclusion chromatography
sTRAIL: Soluble tumor necrosis factor-related apoptosis-inducing ligand
TNF: Tumor necrosis factor
TRAIL: TNF-related apoptosis-inducing ligand
tTFPs: Targeted scTRAIL fusion proteins
