# Supplementary material for: Comparison of antibody-scTRAIL Fc fusion proteins with varying valency for EGFR and TRAIL receptors
Source: Sci Rep. 2025 May 6;15:15801. doi: 10.1038/s41598-025-00476-7 (PMC12056073; doi:10.1038/s41598-025-00476-7)
Supplement: Supplementary file 1 — Supplementary Material 1 [file 41598_2025_476_MOESM1_ESM.docx]

**Supplementary Data**

**Supplementary table 1:** Yields and purity of the produced scTRAIL-antibody fusion proteins.

| **Antibody** | **Molecule #** | **Yield after purification**  **[mg/L supernatant]** | **Purity**  **(% of HMW)** |
| --- | --- | --- | --- |
| **0+2** | | | |
| Fc-scTRAIL-(0+2) | #1 | 4.5 | 0.9 |
| scTRAIL-Fc-(0+2) | #7 | 1.0 | 0.7 |
| **2+2** | | | |
| scFv-Fc-scTRAIL-(2+2) | #4 | 3.8 | 2.5 |
| IgG-scTRAIL-(2+2) | #3 | 1.1 | 0 |
| scTRAIL-Fc-scFv-(2+2) | #9 | 8 | 3.9 |
| **2+1** | | | |
| scFv-Fc-scTRAIL-(2+1) | #6 | 3.2 | 2.9 |
| IgG-scTRAIL-(2+1) | #5 | 1.6 | 4.1 |
| DIgG-Fc-scTRAIL-(0+1) | #2 | 4.2 | 0 |
| **1+1** | | | |
| scFv-scTRAIL-Fc-(1+1) | #11 | 1.3 | 8.3 |
| Fab-scTRAIL-Fc-(1+1) | #10 | 2.6 | 1.2 |
| DFab-scTRAIL-Fc-(0+1) | #8 | 14.0 | 0 |

**Supplementary table 2:** Overview of the cell death induction assay EC_50_ values of the cetuximab blocking experiment for the scTRAIL-antibody fusion proteins on Colo205 cells. CTX: cetuximab n=1; mean of duplicates

| **moieties**  **Fab/scFv + scTRAIL** | **molecule** | **format** | **50% killing [pM]** | **maximal killing [%]** |
| --- | --- | --- | --- | --- |
| **0 + 1** | - | scTRAIL | 778 | 52 |
| **1 + 1** | #10 | Fab-scTRAIL-Fc | 767 | 75 |
| **0 + 1** | #8 | DFab-scTRAIL-Fc | 129 | 100 |
| **1 + 1** | CTX + #10 | CTX + Fab-scTRAIL-Fc | 761 | 100 |

**Supplementary table 3:** One-way ANOVA with Tukey post hoc tests for binding analysis and unpaired *t*-tests for cytotoxicity assays for Colo205 and HCT116 cells.

**Colo205** **HCT116**

**Binding analysis via flow cytometry**

|  | **1+1** (#10, #11) | **2+1** (#5, #6) | **2+2** (#3, #4, #9) |
| --- | --- | --- | --- |
| **0+2** (#1, #7) | **** | **** | **** |
| **1+1** (#10, #11) |  | ns | ns |
| **2+1** (#5, #6) |  |  | ns |

**a)** ANOVA: All formats against each other **b)** ANOVA: All formats against each other

|  | **1+1** (#10, #11) | **2+1** (#5, #6) | **2+2** (#3, #4, #9) |
| --- | --- | --- | --- |
| **0+2** (#1, #7) | **** | **** | **** |
| **1+1** (#10, #11) |  | ns | ns |
| **2+1** (#5, #6) |  |  | ns |

**Cytotoxicity Assays**

|  | **2+2** (#3, #4, #9) |
| --- | --- |
| **0+2** (#1, #7) | **** |

**c)** *t*-test: *Hexavalent* **Targeted** vs **non-targeted d)** *t*-test: *Hexavalent* **Targeted** vs **non-targeted**

|  | **2+2** (#3, #4, #9) |
| --- | --- |
| **0+2** (#1, #7) | **** |

**e)** *t*-test: *Trivalent* **Targeted** vs **non-targeted f)** *t*-test: *Trivalent* **Targeted** vs **non-targeted**

|  | **1+1, 2+1** (10, #11, #5, #6) |
| --- | --- |
| **0+1** (#2, #8) | * |

|  | **1+1, 2+1** (10, #11, #5, #6) |
| --- | --- |
| **0+1** (#2, #8) | ns |

**g)** *t-*test: **Hexavalent** vs **trivalent h)** *t*-test: **Hexavalent** vs **trivalent**

|  | **0+1, 1+1, 2+1** (#2, 8, 10, 11, 5, 6) |
| --- | --- |
| **0+2, 2+2** (#1, 7, 3, 4, 9) | *** |

|  | **0+1, 1+1, 2+1** (#2, 8, 10, 11, 5, 6) |
| --- | --- |
| **0+2, 2+2** (#1, 7, 3, 4, 9) | **** |

**ns** = P > 0.05 ***** = P < 0.05 ****** = P < 0.01 ******* = P < 0.001 ******** = P < 0.0001

*
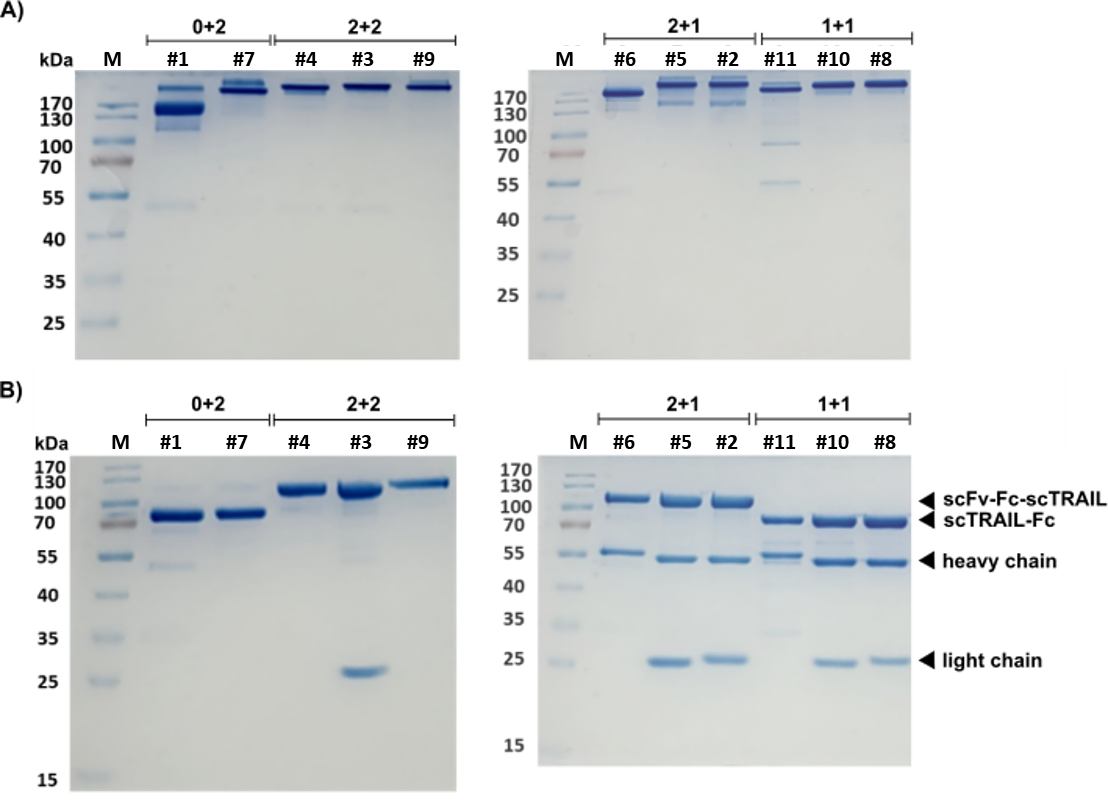
*

**Supplementary Fig. 1: SDS-PAGE (12% PAA) of scTRAIL-antibody fusion proteins. (A)** Non-reducing conditions (4 µg protein/lane) **(B)** reducing conditions (7 µg protein/lane). **M** = marker, **#1** = Fc-scTRAIL-(0+2), **#7** = scTRAIL-Fc-(0+2), **#4** = scFv-Fc-scTRAIL-(2+2), **#3** = IgG-scTRAIL-(2+2), **#9** = scTRAIL-Fc-scFv-(2+2), **#6** = scFv-Fc-scTRAIL-(2+1), **#5** = IgG-scTRAIL-(2+1), **#2** = DIgG-Fc-scTRAIL-(0+1), **#11** = scFv-scTRAIL-Fc-(1+1), #**10** = Fab-scTRAIL-Fc-(1+1), **#8** = DFab-scTRAIL-Fc-(0+1)

**Supplementary Fig. 2**: Size-exclusion chromatography analysis of scTRAIL fusion proteins. Presence of HMW is indicated.

**Supplementary Fig. 3:** EGFR blocking experiment, followed by cell death induction assay of antibody-scTRAIL fusion proteins on Colo205 cells. Titration of antibodies 1:3, CTX (cetuximab) blocking: 100 nM, 30 minutes, 50,000 cells/well, detection with crystal violet staining. n = 1, mean of duplicates.


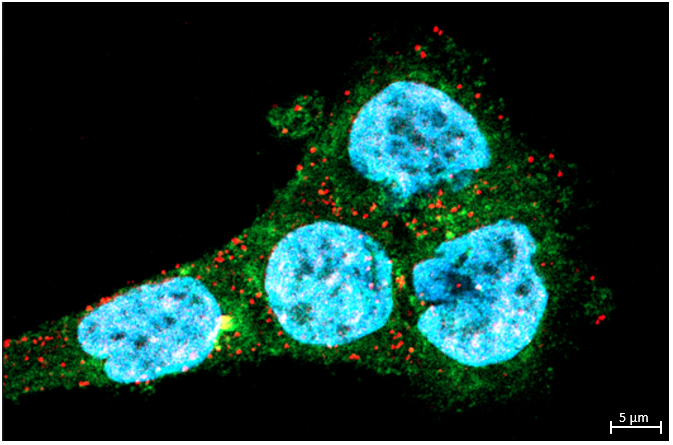


**Supplementary Fig. 4:** Immunofluorescence staining of TRAIL-R1 and -R2 receptors (red) and EGFR (green) studied in HCT116 cells. Cell nucleus was stained with DAPI (blue).
